# Explainable Artificial Intelligence in Mammography: A Systematic Review of Methods, Evaluation Practices, and Clinical Readiness

**DOI:** 10.3390/diagnostics16091412

**Published:** 2026-05-06

**Authors:** Filippo Pesapane, Anna Rotili, Silvia Penco, Valeria Dominelli, Francesca Priolo, Irene Marinucci, Luca Nicosia, Roberto Grasso, Gabriella Pravettoni, Enrico Cassano

**Affiliations:** 1 Breast Imaging Division, Radiology Department, IEO European Institute of Oncology IRCCS, 20141 Milan, Italy; anna.rotili@ieo.it (A.R.); silvia.penco@ieo.it (S.P.); valeria.dominelli@ieo.it (V.D.); francesca.priolo@ieo.it (F.P.); irene.marinucci@ieo.it (I.M.); luca.nicosia@ieo.it (L.N.); enrico.cassano@ieo.it (E.C.); 2 Applied Research Division for Cognitive and Psychological Science, European Institute of Oncology, IRCCS, 20141 Milan, Italy; roberto.grasso@ieo.it (R.G.); gabriella.pravettoni@ieo.it (G.P.); 3 Department of Oncology and Hemato-Oncology, University of Milan, 20122 Milan, Italy

**Keywords:** artificial intelligence, explainable AI, mammography, breast imaging, radiology, trust, explainability

## Abstract

**Background**: Explainable artificial intelligence (XAI) is increasingly proposed to improve trust in mammography-based artificial-intelligence systems, but the validity and clinical readiness of published explanations remain unclear. We aim to systematically review XAI methods applied to mammography and synthesize how explanations are evaluated for validity, robustness, and clinical usefulness. **Methods:** We conducted a systematic review according to PRISMA 2020. MEDLINE/PubMed, Embase, Scopus, Web of Science Core Collection, and the Cochrane Library were searched from 1 January 2015 to 15 January 2026. Two reviewers independently screened records and extracted data; disagreements were resolved by consensus with a third reviewer. Included studies used mammography as the primary input and reported an explicit explanation or interpretability mechanism. Because the literature was methodologically heterogeneous, we performed a structured narrative synthesis and an adapted XAI-specific appraisal of explanation claims, quantitative evaluation, external validation, human-factor assessment, and reporting transparency. **Results**: Fourteen studies were included. Ten studies addressed detection or lesion classification and four addressed risk or outcome prediction. Primary XAI families were interpretable-by-design architectures (6/14), post hoc saliency or attribution methods (5/14), and feature-level explanation methods (3/14). Five studies remained at tier-1 qualitative plausibility only, seven reached tier-2 internal quantitative explanation evaluation, two reached tier-3 external or cross-dataset interpretability assessment, and none reported reader or workflow studies. In the dedicated mammography saliency benchmark, Pointing Game scores for Grad-CAM, Grad-CAM++, and Eigen-CAM ranged from 0.30 to 0.41, indicating only modest lesion-pointing reliability despite acceptable classifier performance. **Conclusions:** Mammography XAI remains dominated by visually plausible explanations that are inconsistently validated. The literature is moving toward task-aligned and intrinsically interpretable designs, yet external validation and clinician-centered evaluation remain rare. Future studies should pre-specify explanation claims, use task-appropriate quantitative metrics, report explanation robustness under distribution shift, and test whether explanations improve human decision-making.

## 1. Introduction

Artificial intelligence (AI) models for mammography have progressed rapidly [1,2,3,4,5,6,7], but the “black-box” nature of many deep learning (DL) systems remains a core barrier to clinical trust, governance, and safe integration [8,9,10]. Saliency maps are image-level visualizations that assign higher intensity to image regions considered more influential for a model prediction. Gradient-weighted class activation mapping (Grad-CAM) is one of the most widely used saliency methods; it back-projects class-specific gradients from a convolutional neural network to generate a course heatmap of regions associated with the predicted class. In contrast, SHapley Additive exPlanations (SHAPs) estimate the contribution of individual features to a prediction, while Local Interpretable Model-agnostic Explanations (LIMEs) approximate model behavior locally with a simpler surrogate model. Because these methods explain different objects (pixels, features, prototypes, or intermediate representations), they should not be evaluated with a single generic criterion. Explainable AI (XAI) in mammography has largely evolved along two methodological trajectories that partially overlap but differ in what they treat as the “explanatory object” [11,12,13,14,15,16,17,18]. The first trajectory is post hoc explanation, in which a model is trained primarily for predictive performance and interpretability is added after training through a separate explanatory mechanism. In mammography, this most commonly takes the form of saliency and attribution techniques that generate heatmaps intended to indicate which image regions are most influenced by a specific prediction, such as class activation map approaches derived from gradients (for example, the Grad-CAM family) [11,13,14,15,18,19,20]. Although these visualizations are attractive because they can be applied to many pre-existing deep networks with minimal changes, evidence from mammography-specific quantitative benchmarking suggests that saliency maps can have only modest lesion-pointing reliability even when the underlying classifier performs acceptably, reinforcing the distinction between visually plausible explanations and faithful localization of the causal evidence [13].

The second trajectory is interpretability by construction, where the model architecture and training objective are deliberately designed so that the reasoning pathway yields intermediate outputs that are intended to be human-interpretable and clinically auditable [21,22,23,24,25,26,27]. This includes high-resolution mammography classifiers that explicitly combine a global module producing a coarse saliency map with a local module operating on selected informative patches, effectively “structuring” the decision into interpretable components rather than generating an explanation solely after the fact. A representative example is the Globally-Aware Multiple Instance Classifier (GMIC) paradigm, which integrates global saliency and local patch evidence to better match the computational constraints and diagnostic logic of large mammograms [28]. In risk prediction settings, interpretability-by-design has also been operationalized through modules that encode clinically meaningful reasoning primitives, such as localized bilateral dissimilarity (an explicit asymmetry-focused mechanism) to approximate the behavior of more complex mammography-based risk models [1,29].

Mammography poses unusually stringent requirements for both approaches, which helps explain why “explanation” is frequently harder here than in many natural-image tasks. Relevant cues may be extremely small and subtle (for instance, fine calcifications), may not form a discrete object (for example, architectural distortion), and may be distributed across multiple foci or views, requiring aggregation across the standard multi-view acquisition and, often, both breasts. In addition, clinically meaningful inference is frequently longitudinal: radiologists compare current images with prior examinations, and both detection and risk models can draw signal from temporal change patterns rather than purely from a single-image snapshot. This makes the expected form of an explanation task-dependent and horizon-dependent; for short-horizon prediction, a lesion-centric explanation may be clinically appropriate, whereas for longer-horizon risk prediction, the model may legitimately rely more on global breast characteristics and asymmetry patterns [30]. This horizon-dependent shift is supported by recent longitudinal interpretability work that analyzes how attributions behave as time-to-cancer varies, underscoring that explanation validity cannot be reduced to a single heatmap criterion across all mammography tasks [31].

The rationale for this review is that explainability in mammography is not a single construct but a family of claims that differ by clinical task, prediction horizon, and explanatory target. We therefore designed the review to answer four specific questions: (1) which XAI families are being used in mammography; (2) what explanatory object each method claims to represent (for example, lesion location, bilateral asymmetry, feature importance, or interpretable intermediate reasoning); (3) how these explanations are evaluated, including whether evaluation is qualitative, quantitative, internal, external, or human-centered; and (4) how close the current evidence base is to clinical readiness. Framing the review in this way avoids conflating visually appealing overlays with validated explanations and provides a more task-aligned synthesis of the field.

The Table 1 summarizes the main terms and their definition in the context of explainable AI.

## 2. Methods

### 2.1. Search Strategy and Information Sources

This systematic review was conducted in accordance with the Preferred Reporting Items for Systematic Reviews and Meta-Analyses (PRISMA) 2020 statement (Supplementary File S1). The review question was focused specifically on explainable and interpretable artificial-intelligence methods applied to mammography for breast cancer detection, lesion classification, and risk or outcome prediction. MEDLINE/PubMed, Embase, Scopus, Web of Science Core Collection, and the Cochrane Library were searched from 1 January 2015 to 15 January 2026. To reduce the risk of missing studies in which explanation was embedded but not explicitly labeled as XAI, the search combined mammography terms with both explicit explainability terms (for example, explainable artificial intelligence, saliency map, attribution map, Grad-CAM, SHAP, and LIME) and structurally related interpretability terms (for example, attention map, prototype, case-based reasoning, weakly supervised localization, and interpretable module). Reference lists of included studies and recent reviews were screened, and forward citation tracking was performed. The complete database-specific search strategies are reported in Supplementary Table S1.

### 2.2. Eligibility Criteria and Study Selection

We included original peer-reviewed studies using mammography or mammography-derived features as the primary input and reporting an explicit explanation or interpretability mechanism with extractable explanation outputs or evaluation. Eligible tasks included cancer detection, lesion classification, risk prediction, and other clinically relevant mammography-based prediction tasks. We excluded non-mammography studies, mixed-modality studies without separable mammography results, papers without a defined explanation output, reviews, editorials, commentaries, conference proceedings, and preprints lacking sufficient methodological detail for reproducible appraisal. Two reviewers independently screened titles and abstracts and then independently assessed the full texts of potentially eligible reports. Disagreements were resolved by consensus, with arbitration by a third reviewer when required. In the revised screening, agreement was substantial at both title/abstract and full-text stages (Cohen’s κ = 0.81 and 0.86, respectively).

Inclusion and exclusion criteria are summarized in Table 2.

### 2.3. Data Extraction

Data were extracted on a piloted standardized form by two reviewers and cross-checked by a senior reviewer. Extracted items included year, setting, mammography modality, clinical task, dataset type, model architecture, XAI family, explanatory target, explanation evaluation method, quantitative metrics, external validation, human-factor assessment, and main methodological limitations. The extraction template should be provided as Supplementary File S2.

### 2.4. Study Appraisal

Because no single validated tool directly captures explanation validity in mammography AI, we used an adapted XAI-specific appraisal framework informed by PROBAST, QUADAS-AI, and prior XAI-evaluation literature. Six domains were scored from 0 to 2: (1) clarity of the explanation claim; (2) alignment between the clinical task and the evaluation target; (3) quantitative explanation evaluation; (4) robustness or external validation; (5) human-factor evaluation; and (6) reporting transparency and reproducibility. Total scores ranged from 0 to 12 and were categorized as having low (0–4), moderate (5–7), or high (8–12) methodological rigor. This appraisal was used to structure the synthesis rather than to exclude studies. The detailed rubric and study-level scores should be provided as Supplementary Table S2. This approach aligns with mammography-specific calls that evaluation frameworks for XAI remain underdeveloped and inconsistently applied [11].

Formal meta-analysis was not attempted because the included studies were heterogeneous in clinical task, explanation target, annotation granularity, and explanation metrics, which made statistical pooling methodologically inappropriate. We therefore performed a structured narrative synthesis supported by quantitative descriptive summaries, using counts and percentages across XAI families, validation tiers, and rigor categories.

**Figure 1:**
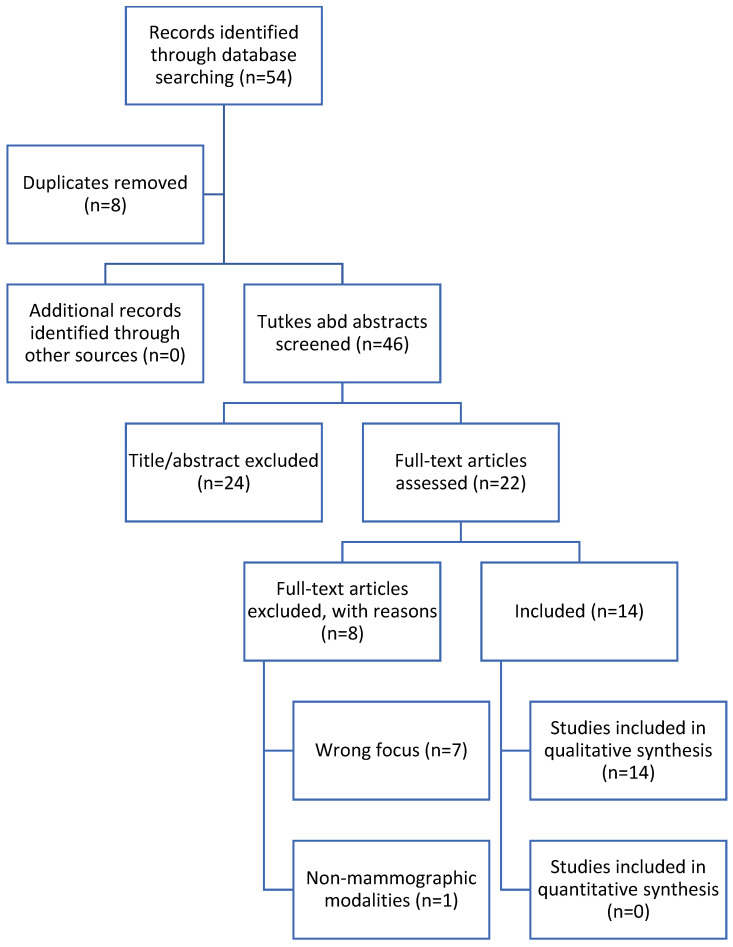
PRISMA flow chart.

Figure 1 summarizes the revised study-selection process. Fifty-four records were identified. After removal of eight duplicates, 46 titles and abstracts were screened. Twenty-two reports underwent full-text assessment, and 14 studies met the inclusion criteria. Seven full-text articles were excluded because they lacked an explicit explanation output or evaluation (n = 3), were non-mammography or mixed-modality studies without separable mammography results (n = 2), were conference proceedings or preprints without sufficient methodological detail (n = 1), or represented overlapping reporting from the same population (n = 1). During revision, one erroneously included digital pathology paper was removed and one eligible mammography paper missed by the original search was added; the final number of included studies therefore remained 14.

## 3. Results

The studies included and their main features are summarized in Table 3. Of the 14 included studies, 10 addressed cancer detection or lesion classification and four addressed risk or outcome prediction. The primary XAI families were interpretable-by-design architectures (6/14), post hoc saliency or attribution methods (5/14), and feature-level explanatory approaches such as SHAP or feature-centric mapping (3/14). Using the revised validation hierarchy, five studies remained at tier 1 (qualitative plausibility only), seven reached tier 2 (internal quantitative explanation evaluation), two reached tier 3 (external or cross-dataset interpretability assessment), and no study reached tier 4 (reader or workflow impact evaluation). Further details are reported in Table 4. Across the evidence base, external explanation validation and clinician-centered evaluation were therefore uncommon.

### 3.1. Taxonomy of XAI Methods Used in Mammography

The dominant mammography-XAI approach remains saliency mapping, particularly Grad-CAM and derivatives (Grad-CAM++, Eigen-CAM), often chosen because it can be applied to existing CNN classifiers with minimal engineering overhead [13]. When lesion localization was explicitly benchmarked, performance was modest for generic saliency methods. In the dedicated mammography benchmark by Cerekci et al. [13], Pointing Game scores for Grad-CAM, Grad-CAM++, and Eigen-CAM were 0.41, 0.30, and 0.35, respectively, increasing only marginally to 0.41, 0.31, and 0.36 among true-positive cases. By contrast, more task-specific pipelines reported higher internal explanation metrics: Camurdan et al. [12] used a ground-truth overlap ratio ranging from 60.26% to 64.18%, and Mellado et al. [22] reported Pointing Game accuracies of 0.6358 for prediction-based maps and 0.5602 for correlation maps. These studies are not directly poolable, but together they show that explanation performance is both method- and task-dependent.

Attention used as a model component or as an augmentation signal: interpretability claims often hinge on “attention highlights ROI,” but rigorous faithfulness validation is frequently absent [37].

Coarse segmentation/explanation maps: approaches that output coarse lesion maps to support interpretability and detection (e.g., CorRELAX framing) [22].

A second cluster designs interpretability into the model. Examples include:

- Global + local MIL-style architectures operating on high-resolution mammograms, producing saliency maps and selecting informative patches as part of the inference pipeline (GMIC line) [34].
- Clinically inspired intermediate maps (e.g., “BIRADS guide map”) that attempt to align internal representations with BI-RADS reasoning [32].
- Explicitly interpretable modules for risk prediction, such as localized bilateral dissimilarity intended to approximate Mirai’s reasoning with an intelligible asymmetry concept [29].
- Prototype/part-based networks that provide example-based interpretability (e.g., pseudo-class part prototype networks), where decisions can be traced to learned prototypes rather than diffuse attributions [33].

For risk and outcome prediction studies, the explanatory target was not uniformly lesion-centric. AsymMirai [29] operationalized localized bilateral dissimilarity as an interpretable intermediate representation, whereas Klanecek et al. [31] showed that attribution patterns changed with time-to-cancer, shifting from focal lesion-like behavior at short horizons to more global breast-characteristic signals at longer horizons. Wang et al. [35] further demonstrated that feature-centric analysis can align learned risk-model features with calcification- and mass-related receptive fields. These findings justify task-aligned evaluation rather than a single universal localization criterion.

### 3.2. Feature-Level Explainability (Radiomics/Learned Features + SHAP/LIME)

For tasks where the model consumes engineered features, radiomics, or compressed representations (including DL-extracted mammographic signatures), SHAP is widely used to rank and directionally interpret feature contributions [36]. This family is most prominent in outcome prediction (recurrence), molecular subtype prediction, and other clinical endpoints, where explanations target covariates rather than pixels.

A distinctive and clinically defining feature of mammography is that decisions are rarely made from a single image in isolation. In routine practice, radiologists integrate priors to determine whether a subtle density is stable, whether an asymmetry is new or evolving, and whether the overall parenchymal pattern is changing in a way that plausibly reflects emerging disease. This “temporal reasoning” is also central for modern mammography-based risk models that predict future cancer rather than simply detecting a present lesion. Models such as Mirai were explicitly developed to forecast 1–5-year risk from screening mammograms, which implies that the signal the model uses is not necessarily lesion-like at all time horizons, but may include distributed, preclinical, or global tissue features that precede overt radiological findings [38].

Within this context, two complementary interpretability strategies have emerged. One is to redesign the model so that the temporal and bilateral cues it uses are intrinsically structured into an interpretable mechanism. A clear example is AsymMirai, which was proposed to approximate Mirai’s short-term risk predictions while embedding an explicit, human-legible module based on localized bilateral dissimilarity—effectively operationalizing the clinically intuitive idea that left–right asymmetry can be informative when it is focal, spatially consistent, and persists or evolves across years [29]. In this approach, interpretability is not merely a post hoc overlay; it is built into the computation as an intermediate representation that can be inspected, audited, and, at least in principle, stress-tested as its own output. The AsymMirai study also emphasizes year-over-year consistency as an interpretability concept, by performing analyses in subgroups where the model’s “reasoning” remained stable across longitudinal examinations, which aligns closely with the way clinicians interpret change over time rather than single-image snapshots [29].

The second strategy acknowledges that many deployed or high-performing risk models will remain complex and therefore applies post hoc longitudinal interpretability analyses to characterize how model explanations evolve as the prediction horizon changes. A representative example is the work by Klanecek et al., who evaluated multiple attribution methods in a longitudinal setting and introduced “attribution heterogeneity” to quantify whether the model’s reliance concentrates on one breast side or reflects broader breast characteristics [31]. Their findings support a clinically consequential interpretation: for short horizons (approximately up to one year), the risk model behaves more like a detection system (with attributions resembling focal, lesion-proximal evidence), while for intermediate horizons (roughly one to three years), it appears to capture early signs that may precede overt detectability, and for longer horizons (beyond three years), the attributions are more consistent with typical breast characteristics such as density-like global features. This is a methodological frontier for mammography XAI because it implies that “the right explanation” is not fixed: a lesion-centric map may be appropriate for near-term detection-like behavior, whereas a breast-characteristic-centric explanation may be legitimate—and potentially more faithful—for longer-term risk prediction, provided the evaluation framework explicitly accounts for the model’s intended time-to-event objective [31].

The most direct mammography-appropriate evaluation checks whether explanation maxima overlap known lesion regions. Cerekci et al. operationalized this using radiologist-drawn boxes and Pointing Game scores, enabling quantitative comparison between saliency methods [13]. This approach is attractive because it yields objective metrics, but it is limited by the imperfect ground truth (boxes vs. segmentation masks) and the possibility that non-lesion contextual cues genuinely contribute to prediction (especially in risk prediction settings).

A notable and underused concept is that interpretability itself should generalize [8]. Moffett et al. explicitly reported that interpretability (activation on relevant lesion portions) persisted across external sites even when AUC for certain subtasks declined, suggesting that interpretability and performance may decouple across domains [23].

Across the mammography XAI literature, structured reader studies that test whether explanations improve radiologist performance, calibration, or error detection are uncommon, and previous mammography-XAI reviews already noted the lack of specialized evaluation frameworks and limited human-confidence evaluation [11].

## 4. Discussion

Across the 14 included studies, the main signal is not simply that mammography XAI exists, but that explanation claims are often broader than the evidence used to support them. Explanations are frequently shown, yet they are less often tested. The most direct mammography-specific saliency benchmark reported Pointing Game scores of only 0.30–0.41 for common Grad-CAM-family methods, demonstrating that visual plausibility does not guarantee faithful lesion localization. This finding should temper claims of clinical readiness for generic saliency overlays.

The review also shows that explanation validity is task-dependent. Detection and lesion-classification studies can reasonably test lesion localization, whereas longer-horizon risk models may be better judged by stability across time, bilateral consistency, or alignment with broader breast characteristics rather than by lesion-box overlapping alone. Interpretable-by-design approaches—such as global–local models, case-based networks, and asymmetry-based modules—are therefore attractive not because they automatically solve explainability, but because they define a clearer explanatory object and a clearer validation target.

The evidence base nevertheless remains limited. Only two included studies reported any external or cross-dataset assessment of interpretability, and no study reported a reader or workflow trial showing that explanations improved radiologist calibration, error detection, or safe reliance. The conclusions of this review should therefore be conservative: mammography XAI is promising, but it is not yet supported by a mature validation culture.

A mammography-specific challenge is the mismatch between the available ground truth and what explanations are asked to demonstrate. Many explanation evaluations rely on radiologist-drawn bounding boxes or similar coarse lesion annotations, but these may not capture the diffuse and textural nature of some mammographic cues, such as architectural distortion, subtle developing asymmetries, or distributed parenchymal patterns. Conversely, in risk prediction tasks—especially when the model’s objective is future cancer risk rather than present lesion detection—an explanation that highlights tissue context outside a lesion box may be legitimate rather than erroneous. Longitudinal interpretability work reinforces this point by showing that attribution patterns can change with time-to-cancer, with shorter horizons behaving more like detection and longer horizons relying more on global breast characteristics, meaning that “lesion-centric localization” cannot be treated as a universal gold standard for explanation validity across mammography tasks [31]. This tension complicates synthesis because different studies may be “right” to evaluate explanations differently, but few papers explicitly justify their evaluation target in relation to the clinical question and prediction horizon.

A further gap arises from the multi-view and bilateral reasoning intrinsic to mammography. Many XAI reports still reduce explanations to single-image overlays, which can under-represent how evidence is aggregated across views and breasts [11,12,13]. Architectures such as GMIC were introduced partly to address mammography’s scale and information-distribution problem by combining a whole-image global module that produces coarse saliency with local high-resolution patches, but even in such designs, explanation reporting is not consistently aligned with the multi-view nature of the clinical workflow [28]. In risk prediction settings, interpretability-by-design approaches that operationalize bilateral asymmetry as an explicit intermediate representation (for example, localized bilateral dissimilarity in AsymMirai) can be read as a direct response to the inadequacy of single-view explanations for a bilateral disease-detection context [29].

External validation represents another underdeveloped axis, and this extends to interpretability itself. Mammography AI has repeatedly shown vulnerability to distribution shift across sites and populations, raising the question of whether explanations remain stable, meaningful, or misleading when models are transported [30]. Only a small subset of studies treat explanation robustness as an explicit endpoint; one notable example is the multi-site external validation of an inherently interpretable lesion model (IAIA-BL), which explicitly examines interpretability behavior across institutions, thereby advancing the notion that interpretability should be evaluated as a transportable property rather than assumed to “come along for free” with performance [23].

Finally, human factors remain a critical but thinly evidenced layer. The central promise of XAI in mammography is not merely to produce explanations, but to improve human–AI interaction: trust calibration, appropriate reliance, and safer decision-making. Yet, structured reader studies that test whether explanations improve radiologist accuracy, reduce overreliance, or help identify AI failure modes are still uncommon, and recent mammography-focused XAI reviews emphasize the lack of specialized evaluation frameworks and the limited clinician-centered validation across the literature [11]. Rather than treating high-performance metrics or visually appealing “explainability” overlays as endpoints in themselves, it is increasingly argued that medical AI should be judged primarily by whether its decision basis is scientifically testable and clinically dependable [8]. In this perspective, post hoc explanations can create an “illusion of understanding” when they remain unvalidated, unstable, or insensitive to the very model they are meant to explain, and strong retrospective performance can still conceal shortcut learning or context-specific signals that collapse under distribution shift. The more reassuring path is therefore not necessarily to demand full transparency, but to accept practical opacity while insisting on disciplined evaluation: preregistered acceptance tests, explicit checks of causal alignment and invariance across scanners/sites/populations, and honest post-deployment monitoring with mechanisms to detect, document, and respond to failures [9]. This shift—from celebrating “explainability” to prioritizing falsifiability, robustness, and real-world surveillance—reframes trustworthy mammography AI as a scientific instrument that must remain reliable under attempted refutation, not merely persuasive in retrospect [8].

Considering the results of this systematic review, some practical recommendations for future mammography XAI studies may be assessed. A first evidence-aligned priority is to pre-specify explanation evaluation with the same discipline applied to model performance metrics, treating explanations as measurable outputs rather than as supporting figures. When lesion localization is an intended claim, authors should adopt objective localization protocols and report them transparently, as in mammography-specific saliency benchmarking using radiologist-defined annotations and explicit metrics [13]. When localization is not the intended claim—particularly for longer-horizon risk prediction—studies should explicitly state what the explanation is expected to represent (for example, global tissue cues, asymmetry, or temporal change) and choose evaluation targets that reflect that objective, rather than defaulting to lesion-centric criteria that may be inappropriate for the task. Longitudinal attribution analyses provide a concrete framework for aligning explanation interpretation with time horizon, and they illustrate why horizon-dependent evaluation should become standard in risk settings [30,31]. A second recommendation is to elevate robustness of explanations across sites, scanners, and populations into a first-class endpoint. Given established generalizability challenges in mammography AI, it is insufficient to show that a model “works” somewhere and that its heatmaps look plausible on a few examples; interpretability itself should be assessed under distribution shift [30]. The IAIA-BL external validation study is instructive here because it explicitly frames interpretability as something that can be evaluated across institutions, setting a precedent for future work to report not only performance drift but also explanation drift [23]. A third recommendation is matching the explanation modality to the clinical question. For detection and diagnostic classification tasks, lesion-centric explanations may be appropriate if they are validated and if their limitations are clearly communicated. For risk prediction, however, explanation frameworks that can express asymmetry, multi-view integration, and longitudinal change are often more conceptually aligned than single-view saliency overlays. This is precisely the design motivation behind interpretable asymmetry-based modules in mammography risk prediction, and it is consistent with evidence that attribution characteristics evolve with prediction horizon [29]. A fourth priority is to incorporate clinician-centered endpoints into evaluation, because the ultimate purpose of XAI in mammography is not to generate visually plausible rationales, but to improve human decision-making in a safety-critical workflow [11]. The field therefore needs evidence that explanations measurably improve calibration (i.e., that radiologists become better at distinguishing when to rely on the model versus when to discount it), reduce inappropriate automation bias, and help clinicians recognize model failure modes and uncertainty in real time. This requirement extends beyond radiologists: mammography screening is a population-facing program, and the perceived legitimacy of AI-supported decisions will be mediated by how clinicians communicate AI use, how accountability is framed, and whether patients feel that the human professional remains in control [39,40]. Studies on the attitudes in breast cancer diagnosis emphasize that trust is shaped by concerns about misdiagnosis, privacy, and responsibility, and that patients tend to prefer AI as an adjunct to—rather than a substitute for—clinical expertise, implying that “explainability” should be evaluated not only as a technical artifact but also as a component of the clinician–patient relationship and shared decision-making [39,40,41]. Accordingly, the next phase of mammography XAI research should pair algorithmic benchmarking with human-centered study designs, incorporating endpoints that capture clinician performance and calibration, communication quality, and downstream trust-related outcomes that ultimately condition whether XAI will be safe and sustainable in practice. A final recommendation is to normalize the reporting of explanation failure modes. Saliency benchmarking work demonstrates that explanation performance can be modest, and reporting these results transparently is clinically valuable because it prevents explanations from being interpreted as reliable lesion localizers when they are not [13]. In mammography, where downstream consequences (recall, biopsy, anxiety) are significant, clarity about when explanations may mislead should be viewed as a safety feature rather than a weakness [3,22,42,43,44,45,46,47,48,49,50].

This review has limitations. First, the eligible evidence base was small, with only 14 included studies after screening. Second, the literature was heterogeneous in clinical tasks, explanatory targets, annotation granularity, and explanation metrics, which precluded formal meta-analysis. Third, conference proceedings were not included in the primary synthesis because they often lacked sufficient methodological detail for reproducible appraisal; this is now acknowledged explicitly as a limitation of the review design. Fourth, because no validated mammography-specific explanation-risk-of-bias tool exists, we used an adapted XAI-specific appraisal framework rather than a formally validated explanation tool. In addition, the current evidence base spans clinically oriented radiology venues and engineering-oriented journals, which contributes to variability in external validation practices, reporting completeness, and clinician-centered evaluation. From a broader scientific-method perspective, this heterogeneity reinforces the need to “measure” and operationalize claims about explanation validity rather than treating explanation visuals as self-evident support for trust, an argument also articulated in contemporary discussions of AI governance and epistemic rigor in radiology [8]. Finally, the review protocol was not prospectively registered; to improve transparency, the revised manuscript should provide the full protocol, extraction template, and appraisal rubric as Supplementary Material.

## 5. Conclusions

In summary, across 14 eligible studies, mammography XAI was most often implemented as either interpretable-by-design model architecture (6/14) or post hoc visual explanation methods (5/14), with a smaller number of feature-level explanation studies (3/14). The field has moved beyond purely illustrative heatmaps, but rigorous explanation validation, external transportability assessment, and reader-centered testing remain uncommon. Progress toward clinically trustworthy mammography XAI will depend on pre-specified explanation claims, task-aligned quantitative metrics, transparent reporting of failure modes, and prospective evaluation of whether explanations improve human decision-making rather than merely increasing the visual persuasiveness of the model output.

## Figures and Tables

**Table 1:** Glossary box.

Term	Definition to Use in the Revised Manuscript
XAI	Explainable artificial intelligence; methods intended to make model reasoning inspectable or interpretable.
SALIENCY MAP	A heatmap highlighting image regions associated with a model prediction.
GRAD-CAM	Gradient-weighted class activation mapping; a class-specific saliency method producing coarse heatmaps from convolutional feature maps.
SHAP	SHapley Additive exPlanations; a feature-attribution method estimating the marginal contribution of each feature.
LIME	Local Interpretable Model-agnostic Explanations; a local surrogate-model approach to explain a prediction.
POINTING GAME	A localization metric testing whether the highest-activation point of an explanation falls inside the lesion annotation.
EXTERNAL VALIDATION	Assessment of the model or explanation on data from a different institution, site, scanner, or population.
READER STUDY	A human-factor study evaluating whether explanations change radiologist performance, calibration, or reliance.

**Table 2:** Eligibility criteria.

Domain	Inclusion Criteria	Exclusion Criteria
Population/data	Mammography-based imaging, including 2D digital mammography and mammography-derived risk or outcome prediction. Contrast-enhanced mammography is eligible only when mammography remains the core modality and the mammography-specific results are separable.	Studies focused exclusively on non-mammography modalities (for example, ultrasound-only, MRI-only, or digital pathology studies).
Intervention	Artificial-intelligence (AI) or machine-learning (ML) model providing an explicit explainability or interpretability mechanism, either post hoc (for example, saliency maps, attribution maps, SHapley Additive exPlanations (SHAPs), or Local Interpretable Model-agnostic Explanations (LIMEs)) or intrinsic (for example, interpretable modules, prototype or case-based networks, or inherently interpretable architectures).	Papers that mention interpretability only in general terms without a defined method or without any explanation output.
Outcomes	Clear description of the explanation method and at least one explanation output or evaluation, such as a heatmap, attribution map, feature-importance plot, prototype, interpretable intermediate representation, localization metric, or clinician-facing interpretability assessment.	Studies without any reported explanation output or with no extractable information about explanation generation or evaluation.
Study type/publication	Original full-text research articles in peer-reviewed journals.	Conference proceedings, abstracts, preprints, reviews, editorials, and commentaries. Conference proceedings may be mentioned in a sensitivity note but should not enter the primary synthesis unless full methodological detail is available.

**Table 3:** Studies included.

Study	Clinical Task	Primary XAI Family	Explanatory Target	Explanation Evaluation to Report
Cerekci et al., 2024 (Eur J Radiol) [13]	Cancer detection	Post hoc saliency (Grad-CAM, Grad-CAM++, Eigen-CAM)	Lesion localization	Pointing Game against radiologist boxes; scores 0.30–0.41.
Kim et al., 2018 (Phys Med Biol) [32]	Mass classification	Intrinsic BI-RADS-guided network	BI-RADS-like guide map	Qualitative visualization aligned with lesion-characterization logic.
Choukali 2024 (Sci Rep) [33]	Mass-lesion classification	Case-based interpretable deep learning (IAIA-BL)	Relevant lesion parts and semantic mass margins	Case-based explanations and comparison against black-box attention; no accuracy loss relative to a black-box baseline.
Shen et al., 2021 (Med Image Anal) [34]	Screening classification	Global-local weakly supervised classifier	Saliency map and informative patches	Internal localization and patch-selection interpretability with cross-dataset evaluation.
Donnelly et al., 2024 (Radiology) [29]	1–5-year risk prediction	Interpretable asymmetry module (AsymMirai)	Localized bilateral dissimilarity	Correlation with Mirai and longitudinal consistency analyses.
Wang et al., 2025 (Radiology: AI) [35]	Risk prediction/feature characterization	Feature-centric XAI pipeline	Receptive fields and mammographic lesion features	Correlation of risk-model features with calcification- and mass-related locations; task-aligned performance comparison.
Klanecek et al., 2024 (Phys Med Biol) [31]	Risk prediction	Longitudinal attribution analysis	Horizon-dependent attribution patterns	Attribution heterogeneity across time-to-cancer.
Moffett et al., 2025 (PLOS ONE) [23]	Lesion malignancy prediction	External validation of IAIA-BL	Activation on relevant lesion regions	Multi-site external validation of interpretability and performance.
Camurdan et al., 2025 (Insights Imaging) [12]	Cancer detection	Patch-based curriculum model with Grad-CAM	Lesion-focused heatmaps	Ground-truth overlap ratio plus external dataset testing.
Talaat et al., 2024 (Cancers) [16]	Mammogram classification	Post hoc Grad-CAM	Heatmap highlighting of cancer regions	Qualitative explanation only.
Acosta-Jiménez et al., 2025 (Diagnostics) [20]	Lesion classification	SHAP-based relevance mapping across DM and CESM	Pixel-level relevance maps	Qualitative anatomical coherence across modalities; no dedicated explanation metric.
Sha et al., 2025 (Front Immunology) [36]	Recurrence prediction	SHAP feature attribution	Feature-contribution ranking	SHAP summary of mammographic and clinicopathologic features; explanation claim not independently validated.
Mellado et al., 2025 (Front Oncology) [22]	Lesion detection	CorRELAX feature attribution	Coarse correlation maps	Pointing Game 0.5602–0.6358 on VinDr-Mammo.
Lopez et al., 2024 (Pattern Recogn. Lett.) [37]	Benign vs. malignant classification	Attention-map augmentation (PHAM)	Attention-guided ROI emphasis	Attention maps used as conditioning signal; qualitative interpretability only.

**Table 4:** Hierarchy of explanation-validation rigor across included studies.

Study	Quantitative Explanation Metric	External/Cross-Dataset Interpretability Assessment	Reader Study	Highest Validation Tier
Cerekci et al., 2024 (Eur J Radiol) [13]	Yes: Pointing Game	No	No	Tier 2
Kim et al., 2018 (Phys Med Biol) [32]	No	No	No	Tier 1
Choukali 2024 (Sci Rep) [33]	Internal interpretability comparison	No	No	Tier 2
Shen et al., 2021 (Med Image Anal) [34]	Internal localization/patch interpretability	Partial cross-dataset assessment	No	Tier 3
Donnelly et al., 2024 (Radiology) [29]	Task-aligned quantitative correlation/consistency	No	No	Tier 2
Wang et al., 2025 (Radiology: AI) [35]	Feature-centric quantitative characterization	No	No	Tier 2
Klanecek et al., 2024 (Phys Med Biol) [31]	Yes: attribution heterogeneity	No	No	Tier 2
Moffett et al., 2025 (PLOS ONE) [23]	Yes: activation on relevant lesion portions	Yes: multi-site external validation	No	Tier 3
Camurdan et al., 2025 (Insights Imaging) [12]	Yes: ground-truth overlap ratio	External performance testing only	No	Tier 2
Talaat et al., 2024 (Cancers) [16]	No	No	No	Tier 1
Acosta-Jiménez et al., 2025 (Diagnostics) [20]	No dedicated explanation metric	No	No	Tier 1
Sha et al., 2025 (Front Immunology) [36]	No dedicated validation of explanation claim	No	No	Tier 1
Mellado et al., 2025 (Front Oncology) [22]	Yes: Pointing Game	No	No	Tier 2
Lopez et al., 2024 (Pattern Recogn. Lett.) [37]	No	No	No	Tier 1

## Data Availability

No new data were created or analyzed in this study. Data sharing is not applicable to this article.

## Supplementary Materials

The following supporting information can be downloaded at: https://www.mdpi.com/article/10.3390/diagnostics16091412/s1. Supplementary File S1: PRISMA checklist. Table S1: Database-specific search strategies and retrieval yields, Supplementary File S2: Standardized data-extraction template, Table S2: Adapted XAI appraisal rubric and study-level scores. Reference [51] is cited in Supplementary File S1.
