# Supplementary material for: Explainable Artificial Intelligence in Mammography: A Systematic Review of Methods, Evaluation Practices, and Clinical Readiness
Source: Diagnostics (Basel). 2026 May 6;16(9):1412. doi: 10.3390/diagnostics16091412 (PMC13163533; doi:10.3390/diagnostics16091412)
Supplement: Supplementary file 1 [file diagnostics-16-01412-s001.zip › Supplementary File S2.pdf]

Supplementary Materials

Supplementary Table S1. Database-specific search strategies and retrieval yields

The table below summarizes the database-level retrieval counts used to match the revised PRISMA diagram. Exact database-specific search strings are provided immediately afterwards.

Database	Date searched	Records	Notes
MEDLINE/PubMed	15 Jan 2026	17	Database search only; title/abstract strategy with publication-date filter.
Embase	15 Jan 2026	14	EMTREE-aware title/abstract/keyword strategy; article limit applied.
Scopus	15 Jan 2026	13	TITLE-ABS-KEY strategy with year limit 2015–2026.
Web of Science Core Collection	15 Jan 2026	9	Topic search (TS) across Core Collection indexes.
Cochrane Library	15 Jan 2026	1	Keyword search in title/abstract/keyword fields; no review articles included in final synthesis.
Total before de-duplication	—	54	Consistent with the revised PRISMA flow in the manuscript.

MEDLINE/PubMed

```
(
  (mammograph*[Title/Abstract] OR mammogram*[Title/Abstract] OR mammographic[Title/Abstract]
   OR "digital mammography"[Title/Abstract] OR "screening mammography"[Title/Abstract])
  AND
  (
    "explainable artificial intelligence"[Title/Abstract] OR "explainable AI"[Title/Abstract]
    OR XAI[Title/Abstract] OR explainab*[Title/Abstract] OR interpretab*[Title/Abstract]
    OR "saliency map"[Title/Abstract] OR "attention map"[Title/Abstract]
    OR "attribution map"[Title/Abstract] OR "class activation map"[Title/Abstract]
    OR "Grad-CAM"[Title/Abstract] OR "Grad-CAM++"[Title/Abstract] OR "Eigen-CAM"[Title/Abstract]
    OR SHAP[Title/Abstract] OR LIME[Title/Abstract] OR prototype*[Title/Abstract]
    OR "case-based reasoning"[Title/Abstract] OR "weakly supervised localization"[Title/Abstract]
    OR "interpretable module"[Title/Abstract] OR "feature attribution"[Title/Abstract]
  )
)
AND ("2015/01/01"[Date - Publication] : "2026/01/15"[Date - Publication])
```

Embase

```
(
  ('mammography'/exp OR mammograph*:ti,ab,kw OR mammogram*:ti,ab,kw OR mammographic:ti,ab,kw)
  AND
  (
    'explainable artificial intelligence':ti,ab,kw OR 'explainable ai':ti,ab,kw OR xai:ti,ab,kw
    OR explainab*:ti,ab,kw OR interpretab*:ti,ab,kw OR 'saliency map':ti,ab,kw
    OR 'attention map':ti,ab,kw OR 'attribution map':ti,ab,kw OR 'class activation map':ti,ab,kw
    OR 'grad-cam':ti,ab,kw OR 'grad-cam++':ti,ab,kw OR 'eigen-cam':ti,ab,kw
    OR shap:ti,ab,kw OR lime:ti,ab,kw OR prototype*:ti,ab,kw OR 'case-based reasoning':ti,ab,kw
    OR 'weakly supervised localization':ti,ab,kw OR 'interpretable module':ti,ab,kw
    OR 'feature attribution':ti,ab,kw
  )
)
AND [2015-2026]/py
AND [article]/lim
```

Scopus

```
TITLE-ABS-KEY (
  (mammograph* OR mammogram* OR mammographic OR "digital mammography" OR "screening mammography")
  AND
  (
    "explainable artificial intelligence" OR "explainable ai" OR xai OR explainab* OR interpretab*
    OR "saliency map" OR "attention map" OR "attribution map" OR "class activation map"
    OR "grad-cam" OR "grad-cam++" OR "eigen-cam" OR shap OR lime OR prototype*
    OR "case-based reasoning" OR "weakly supervised localization" OR "interpretable module"
    OR "feature attribution"
  )
)
AND PUBYEAR > 2014
AND PUBYEAR < 2027
```

Web of Science Core Collection

```
TS=(
  (mammograph* OR mammogram* OR mammographic OR "digital mammography" OR "screening mammography")
  AND
  (
    "explainable artificial intelligence" OR "explainable ai" OR xai OR explainab* OR interpretab*
    OR "saliency map" OR "attention map" OR "attribution map" OR "class activation map"
    OR "grad-cam" OR "grad-cam++" OR "eigen-cam" OR shap OR lime OR prototype*
    OR "case-based reasoning" OR "weakly supervised localization" OR "interpretable module"
    OR "feature attribution"
  )
)
Refined by: DOCUMENT TYPES = (ARTICLE)
Timespan: 2015-01-01 to 2026-01-15
```

Cochrane Library

```
(
  ([mh Mammography] OR mammograph*:ti,ab,kw OR mammogram*:ti,ab,kw OR mammographic:ti,ab,kw)
  AND
  (
    "explainable artificial intelligence":ti,ab,kw OR "explainable ai":ti,ab,kw OR xai:ti,ab,kw
    OR explainab*:ti,ab,kw OR interpretab*:ti,ab,kw OR "saliency map":ti,ab,kw
    OR "attention map":ti,ab,kw OR "attribution map":ti,ab,kw OR "class activation map":ti,ab,kw
    OR "grad-cam":ti,ab,kw OR "grad-cam++":ti,ab,kw OR "eigen-cam":ti,ab,kw
    OR shap:ti,ab,kw OR lime:ti,ab,kw OR prototype*:ti,ab,kw
    OR "case-based reasoning":ti,ab,kw OR "weakly supervised localization":ti,ab,kw
    OR "interpretable module":ti,ab,kw OR "feature attribution":ti,ab,kw
  )
)
Publication window: 1 January 2015 to 15 January 2026
```

Supplementary File S2. Standardized data-extraction template

The extraction form below was used after pilot testing on three studies. Fields were completed independently by two reviewers and reconciled by consensus. The final column states how each field was to be interpreted during extraction to improve consistency.

Domain	Field	Type	Extraction guidance
Identification	Study ID	Text	Unique label used throughout screening and extraction (e.g., first author + year).
Identification	Full citation	Text	Journal article citation used in the manuscript reference list.
Identification	Country / setting	Text	Country and whether the dataset was single-center, multi-center, public, or mixed.
Design	Study design	Categorical	Retrospective development, retrospective validation, benchmark study, or methodological study.

Domain	Field	Type	Extraction guidance
Population / data	Mammography modality	Categorical	2D digital mammography, synthesized 2D from DBT, contrast-enhanced mammography, or other mammography-derived input.
Population / data	Unit of analysis	Categorical	Image, breast, lesion, exam, or patient.
Population / data	Sample size	Numeric + text	Record number of women, exams, images, and lesions when separately reported.
Population / data	Dataset provenance	Categorical + text	Public dataset, institutional dataset, multi-site dataset, or externally validated cohort.
Population / data	Annotation granularity	Categorical	Image-level labels, lesion boxes, lesion masks, descriptors, or none.
Task	Clinical task	Categorical	Detection, lesion classification, screening classification, risk prediction, recurrence prediction, or biomarker prediction.
Task	Target outcome	Text	Primary endpoint predicted by the model (e.g., malignant vs benign, 1–5 year risk).
Model	Model family / architecture	Text	CNN, transformer, weakly supervised network, prototype network, feature-based ML, etc.
Model	Primary comparator	Text	Black-box baseline, alternate saliency method, radiologist benchmark, or none.
Explanation	XAI family	Categorical	Post hoc saliency, feature attribution, attention map, prototype/case-based, or intrinsically interpretable design.

Continuation of extraction template

Domain	Field	Type	Extraction guidance
Explanation	Explanation target	Text	What the explanation is intended to explain: lesion location, semantic feature, bilateral asymmetry, feature contribution, etc.
Explanation	Explanation claim	Text	Explicit claim made by the authors about what the explanation demonstrates.
Evaluation	Explanation evaluation type	Categorical	Qualitative plausibility, internal quantitative evaluation, external interpretability validation, reader study, or mixed.
Evaluation	Quantitative explanation metrics	Text	Pointing Game, overlap ratio, attention agreement, attribution stability, feature ranking, or task-aligned proxy metrics.
Evaluation	Performance metrics	Text	AUC, sensitivity, specificity, AP, calibration, or other predictive metrics reported alongside explanation analyses.
Evaluation	Robustness / external validation	Text	Cross-dataset testing, subgroup analysis, temporal stability, sensitivity analysis, or none.
Human factors	Clinician-facing assessment	Text	Reader study, expert scoring, qualitative expert review, workflow evaluation, or none.
Reporting	Data / code transparency	Text	Availability of code, pretrained weights, public dataset details, split definitions, and reproducibility information.
Reporting	Conflicts / funding	Text	Funding source and any author-reported competing interests.
Synthesis	Key limitations	Free text	Main threats to explanation validity or clinical readiness extracted by the reviewers.
Synthesis	Reviewer notes	Free text	Structured notes used to assign validation tier and appraisal score.

Supplementary Table S2. Adapted XAI appraisal rubric and study-level scores

Part A defines the consensus scoring rubric. Part B reports study-level scores for the 14 included articles. These scores are author-applied synthesis judgments used to structure the revised qualitative review; they are not reported by the original primary studies themselves.

Rubric used for study appraisal

Domain	Score 0	Score 1	Score 2
D1. Clarity of the explanation claim	No explicit statement of what the explanation is intended to show.	Explanation purpose is implied but incompletely specified.	Explanation claim is explicit, specific, and clinically interpretable.
D2. Alignment between task and evaluation target	Explanation output is not meaningfully aligned with the clinical or technical task.	Partial alignment, usually via qualitative examples or indirect proxies.	Direct alignment between explanation target and the claimed task (e.g., lesion localization against lesion reference data).
D3. Quantitative explanation evaluation	No quantitative explanation evaluation.	Limited or proxy quantitative assessment without a clearly matched reference standard.	Explicit quantitative evaluation with a task-appropriate metric or comparator.
D4. Robustness / external validation	No robustness, sensitivity, or external interpretability assessment.	Internal sensitivity analysis, subgroup assessment, or partial robustness analysis only.	External dataset testing or clearly reported cross-dataset interpretability assessment.
D5. Human-factor evaluation	No clinician-facing assessment.	Structured expert review or human-comparison analysis without a reader/workflow study.	Formal reader study, workflow study, or measured impact on human decision-making.
D6. Reporting transparency and reproducibility	Insufficient reporting to reproduce core design or explanation analysis.	Adequate reporting of data, splits, methods, and metrics.	Strong transparency, including reproducible splits and clearly described explanation implementation; code or reusable assets available when applicable.

Study-level appraisal scores

Study	D1	D2	D3	D4	D5	D6	Total	Rigor	Tier
Cerekci et al., 2024	2	2	2	0	0	1	7	Moderate	Tier 2
Kim et al., 2018	1	1	0	0	0	1	3	Low	Tier 1
Barnett et al., 2021	2	2	1	0	0	2	7	Moderate	Tier 2
Shen et al., 2021	2	2	1	2	0	2	9	High	Tier 3
Donnelly et al., 2024	2	2	1	1	0	1	7	Moderate	Tier 2
Wang et al., 2025	2	2	1	1	0	1	7	Moderate	Tier 2
Klanecek et al., 2024	2	2	1	1	0	1	7	Moderate	Tier 2
Moffett et al., 2025	2	2	2	2	0	2	10	High	Tier 3
Camurdan et al., 2025	2	2	1	1	0	1	7	Moderate	Tier 2
Talaat et al., 2024	1	1	0	0	0	1	3	Low	Tier 1
Acosta-Jimenez et al., 2025	1	1	0	0	0	1	3	Low	Tier 1

Study	D1	D2	D3	D4	D5	D6	Total	Rigor	Tier
Sha et al., 2025	1	1	0	0	0	1	3	Low	Tier 1
Mellado et al., 2025	2	2	2	0	0	1	7	Moderate	Tier 2
Lopez et al., 2024	1	1	0	0	0	1	3	Low	Tier 1

Domain abbreviations: D1, clarity of explanation claim; D2, task–evaluation alignment; D3, quantitative explanation evaluation; D4, robustness or external validation; D5, human-factor evaluation; D6, reporting transparency and reproducibility.

Validation tiers used in the revised manuscript: Tier 1, qualitative plausibility only; Tier 2, internal quantitative explanation evaluation; Tier 3, external or cross-dataset interpretability assessment.

.
